# Implications of empirical testing and treatment for atypical bacteria in patients hospitalized with COVID, influenza, or RSV: a retrospective observational cohort study

**DOI:** 10.1007/s10096-026-05405-8

**Published:** 2026-01-17

**Authors:** Bo Langhoff Hønge, Jacob Redder, Thomas Greve, Lars Skov Dalgaard, Anita Rath Sørensen, Lotte Ebdrup, Rajesh Mohey, Britta Tarp, Mette Holm, Lars Østergaard, Merete Storgaard

**Affiliations:** 1 Department of Infectious Diseases, Aarhus University Hospital, Palle Juul- Jensens Boulevard 99, Aarhus, 8200 Denmark; 2 Department of Data and Data Utilization, Central Denmark Region, Aarhus, Denmark; 3 Department of Clinical Microbiology, Aarhus University Hospital, Aarhus, Denmark; 4 Department of Internal Medicine, Gødstrup Hospital, Herning, Denmark; 5 Department of Internal Medicine, Horsens Regional Hospital, Horsens, Denmark; 6 Department of Internal Medicine, Randers Regional Hospital, Randers, Denmark; 7 Department of Internal Medicine, Viborg Regional Hospital, Viborg, Denmark; 8 Medical Diagnostic Center, University Clinic for Innovative Patient Pathways, Regional Hospital Central Jutland, Silkeborg, Denmark; 9 Department of Pediatrics and Adolescent Medicine, Aarhus University Hospital, Aarhus, Denmark

**Keywords:** Registries, Epidemiology, Legionella, Mycoplasma, Chlamydia, Denmark

## Abstract

**Purpose:**

Patients admitted with viral respiratory tract infections are at risk ofbacterial co-infections that may exacerbate disease severity. Detection of atypical bacteria requires specific laboratory diagnostic modality and specific antibiotics.

**Methods:**

In this retrospective regionwide cohort study we included all patients admitted to a hospital in the Central Denmark Region with COVID-19, influenza A, influenza B, or Respiratory Syncytial Virus (RSV) from February 2019 to February 2024. Firstly, we investigated the number of patients testing positive for atypical bacterial co-infection. Secondly, we evaluated associations with diagnostic testing for these atypical bacteria, and the use and associations with administration of empirical treatment with clarithromycin.

**Results:**

During the study period a total of 19,651 patients were admitted with one of the viral respiratory tract infections. Only 21 patients tested positive for atypical bacterial co-infection, corresponding to 0.1% of those tested (*n* = 2,369). Empirical clarithromycin was administered to 859 (4.4%) patients. Still 17 out of the 21 patients (81.0%) with atypical bacterial co-infection did not receive clarithromycin before the result of diagnostic test was available.

**Conclusions:**

Our findings do not support routine testing for atypical bacterial co-infection and use of empirical treatment for atypical pneumonia in this population.

## Introduction

Respiratory viral infections such as influenza, respiratory syncytial virus (RSV), and corona virus disease 2019 (COVID-19) are a major cause of global health burden [1, 2]. Due to restrictions imposed during the emergence of the SARS-CoV-2 epidemic, societies experienced a reduction in incidence of other respiratory infections. Since then, we have seen a surge in both influenza and RSV cases and in the 2022/23 and 2023/24 winter seasons all three viruses were co-circulating in Europe [3, 4].

While respiratory viruses alone can be deleterious; bacterial co-infections may exacerbate disease severity, prolong hospitalization, and increase risk of mortality [5, 6]. *Legionella pneumophila*, *Mycoplasma pneumonia*, *Chlamydia pneumonia*, and *Chlamydia psittaci* are often referred to as atypical bacteria due to structural and metabolic characteristics. As they are intracellular pathogens they cannot be diagnosed by standard bacterial culture, and they are not susceptible to antimicrobials targeting the bacterial cell wall such as penicillin [7]. For community-acquired pneumonia with an atypical pathogen many guidelines recommend treatment with a macrolide such as clarithromycin [8, 9].

In this study we investigated the proportion of atypical bacterial co-infections in patients admitted with a verified viral respiratory infection. Furthermore, we evaluated the proportion and associations of testing for atypical bacterial co-infection, and the use of empirical clarithromycin.

## Materials and methods

This study cohere to the STROBE guidelines for reporting methodological approach [10].

### Study design

Retrospective observational cohort study.

### Setting

The study was conducted in the Central Denmark Region which has a population of 1.3 million people corresponding to 23% of the Danish population [11]. All patients in this region with medical conditions requiring acute hospital evaluations are serviced by public (tax paid) hospitals placed in the following cities; Aarhus, Horsens, Gødstrup, Randers, Viborg, and Silkeborg. Pediatric departments are situated in Aarhus, Gødstrup, Randers, and Viborg. Previously hospitals were also situated in Herning and Holstebro, but in February 2022 these two hospitals merged into Gødstrup Hospital. The study period for this study was February 3rd 2019 to February 3rd 2024.

### Participants

We included all patients admitted to a hospital in the Central Denmark Region who had a positive test for either SARS-CoV-2, influenza A and B, or RSV within 24 h before admission up to 48 h after time of admission. This resembles participation criteria from a recent Swedish study [6]. Test results from samples originating from both hospitals and the primary sector were included.

### Ethics

The study was approved as a quality assurance project by the administrative units covering each contributing hospital. Patient´s civil registration number (CPR) were pseudonymized by the regional business intelligence unit (Department of Data and Data Utilization) and uploaded to a secure server platform available to the investigators only. In Denmark ethics approval are not required for registry-based quality assurance projects.

### Variables

Primary outcome was the proportion of patients testing positive for an atypical bacterial co-infection with *Legionella pneumophilia*, *Mycoplasma pneumonia*, *Chlamydia psittaci*, or *Chlamydia pneumonia* within 24 h before and 168 h (7 days) after time of admission and counting to time of sampling for atypical bacterial agent.

Secondary outcomes were the proportion of patients which were tested and not tested for atypical bacterial co-infection of the above mentioned species, and clinical associations to test application. Additional secondary outcomes were the proportion of patients receiving empirical treatment with clarithromycin against presumed co-infection, and clinical associations with use of clarithromycin. According to local guidelines during the study period clarithromycin was the recommended empirical treatment for suspected pneumonia due to atypical bacteria. Empirical use was defined as administration of clarithromycin within 48 h of admission and before test results were available for those patients tested for atypical bacterial co-infection. Penicillin allergy was defined as any registration of allergic reaction to any drug containing penicillins.

The study population was described by viral agent, admission hospital, time of admission sex and age. First-measured vital parameters (temperature, systolic blood pressure, and respiratory rate) and first-measured laboratory test results (leukocyte count, C-reactive protein (CRP), alanine transaminase (ALT), creatinine, and urea) were selected as relevant clinical parameters.

### Microbiology

Since 2017 PCR-based point-of-care tests (POCT) have been available at all hospitals in the Central Denmark Region for testing of influenza A and influenza B. SARS-CoV-2 and RSV was added in the POCT in 2020 [12]. In addition conventional PCR tests could be prescribed, and test results for both analyses were pooled into one variable for this study.

Department of Clinical Microbiology, Aarhus University Hospital, is the only microbiological laboratory in the Central Denmark Region and handles analyses from all hospitals and the primary sector. The “atypical pneumonia” package contains dual PCR testing for *Legionella pneumophila* and *Mycoplasma pneumonia*. Physicians may also request specific *Legionella* or *Mycoplasma* single PCR. The “atypical pneumonia” package and the specific *Legionella pneumophila* PCR are only performed on samples from the lower respiratory tract such as expectorate, tracheal suction, or fluid from a bronchoalveolar lavage. *Mycoplasma pneumonia* PCR could be done on either lower respiratory tract samples or throat swabs when lower respiratory material was unavailable. Throat swabs were not used for *Legionella pneumophilia* PCR, which was restricted to lower respiratory tract specimens. The ImmuView assay (SSI Diagnostica, Hillerød, Denmark) is a urine-based combi-test detecting *Streptococcus pneumonia* and *Legionella pneumophila* antigen, however this test is only performed on patients admitted to the intensive care wards or upon special agreement with the laboratory. *Chlamydia psittaci* and *pneumonia* PCR were performed separately, but results were pooled here for simplicity.

### Data sources/measurements

All hospitals in the Central Denmark Region use the same electronic healthcare record platform for patient care (Elektronisk patientjournal (EPJ), Systematic A/S, Aarhus, Denmark). Data are centralized at the regional business intelligence unit (Department of Data and Data Utilization) located in Aarhus. In this system all data on laboratory results, medication lists et cetera are securely stored. Data was extracted based on pre-defined study aims.

For laboratory analyses separate time variables were stored; time of ordering the analysis, time of sample reception at the laboratory, and time of publishing the analysis result. The time variables used are stated when applicable. For pharmaceuticals we chose to use the time of drug administration because time of prescription may precede actual drug administration significantly.

## Study size

A sample size calculation was not performed before initiating this study. The final sample size was based on all admissions in the Central Denmark Region fulfilling the inclusion criteria. In Denmark quality assurance projects allows for retrospective analyses during a five-year period which determined the study period and thereby the sample size.

### Statistical methods

Demographic and clinical characteristics were stratified and summarized by descriptive statistics. Proportion of patients co-infected with atypical bacteria was presented in absolute numbers and as a percentage of the study population. Associations with being tested for atypical bacteria and having clarithromycin administered were calculated with logistic regression according to the recommendations from the journal [13]. We performed both an univariate and sex- and age-adjusted analyses and estimates were presented as odds ratios (ORs) and adjusted ORs (aORs) with 95% confidence intervals (95% CIs). In the regression analysis separate patient groups were created for patients with missing information on clinical parameters. To account for potential differences in disease severity at different hospitals in the region, we additionally performed a multivariate analysis further adjusting for clinical parameters when estimating these associations with individual hospitals. Aarhus University Hospital was chosen as the reference group in the regression analyses due to this hospital being the referral tertiary hospital in the region and having the largest number of cases. When evaluating continuous parameters associated with atypical bacterial co-infection we assessed for normal distribution and continued with Wilcoxon ranksum tests (non-normal distribution). STATA version 18.5 (College Station, Texas, USA) was used for all statistical analyses.

## Results

During the study period a total of 19,651 patients were admitted with one of the viral respiratory infections (10,773 COVID, 3,867 influenza A, 521 influenza B, 4,224 RSV, and 266 with multiple virus). Patient characteristics are presented in Table 1. Overall, 2,262 (11.5%) were tested for Legionella, 2,091 with the “atypical pneumonia package”, 50 with *Legionella pneumophila* PCR, and 121 with a urine antigen test. The seven (0.3%) legionella positive patients were all detected with the “atypical pneumonia package”. Mycoplasma testing was performed for 2,360 (12.0%) patients; 2,099 with the “atypical pneumonia package”, and 261 with the *Mycoplasma pneumonia* PCR. Of the 14 (0.6%) Mycoplasma positive patients, four cases were identified by the “atypical pneumonia package” and 10 with the *Mycoplasma pneumonia* PCR. Only 180 (0.9%) of the patients had been tested for *Chlamydia pneumonia* or *psittaci* and all test results were negative. Testing patterns are presented in Table 2.

**Table 1:** Descriptive demographic information by virus type

	SARS-CoV-2, *n* (%)	Influenza A, *n* (%)	Influenza B, *n* (%)	RSV, *n* (%)	Multiple virus	In total, *n*
	*n* = 10,773	*n* = 3,867	*n* = 521	*n* = 4,224	*n* = 266	*n* = 19,651
Hospital (city)						
Aarhus	2,849 (59.2)	907 (18.8)	117 (2.4)	876 (18.2)	64 (1.3)	4,813
Horsens	1,215 (63.6)	465 (24.4)	35 (1.8)	180 (9.4)	15 (0.8)	1,910
Gødstrup	2,581 (58.3)	827 (18.7)	117 (2.6)	846 (19.1)	59 (1.3)	4,430
Randers	1,920 (49.5)	717 (18.5)	120 (3.1)	1,055 (27.2)	65 (1.7)	3,877
Silkeborg/Viborg	2,208 (47.8)	951 (20.6)	132 (2.9)	1,267 (27.4)	63 (1.4)	4,621
Year of admission*						
2019	0 (0.0)	691 (73.7)	19 (2.0)	221 (23.6)	7 (0.8)	938
2020	982 (52.9)	494 (26.6)	18 (1.0)	337 (18.1)	27 (1.5)	1,858
2021	1,170 (48.2)	13 (0.5)	9 (0.4)	1,224 (50.4)	11 (0.5)	2,427
2022	5,684 (66.8)	1,244 (14.6)	32 (0.4)	1,453 (17.1)	98 (1.2)	8,511
2023	2,785 (54.8)	940 (18.5)	443 (8.7)	817 (16.1)	99 (2.0)	5,084
2024	152 (18.3)	485 (58.2)	0 (0.0)	172 (20.7)	24 (2.9)	833
Sex						
Female	5,062 (53.7)	1,943 (20.6)	270 (2.9)	2,027 (21.5)	124 (1.3)	9,426
Male	5,711 (55.9)	1,924 (18.8)	251 (2.5)	2,197 (21.5)	142 (1.4)	10,224
Age, years						
0–17	1,124 (22.3)	724 (14.3)	254 (5.0)	2,830 (53.1)	113 (2.2)	5,045
18–65	3,625 (62.1)	1,484 (25.4)	230 (3.9)	434 (7.4)	64 (1.1)	5,837
65+	6,024 (68.7)	1,659 (18.9)	37 (0.4)	960 (11.0)	89 (1.0)	8,769

*From February 3rd, 2019 to February 3rd, 2024

**Table 2:** Atypical bacteria testing pattern by virus

	SARS-CoV-2, *n* (%)	Influenza A, *n* (%)	Influenza B, *n* (%)	RSV, *n* (%)	Multiple virus	In total, *n* (%)
	*n* = 10,773	*n* = 3,867	*n* = 521	*n* = 4,224	*n* = 266	*n* = 19,651
Legionella						
Positive	4 (0.0)	2 (0.0)	0 (0.0)	1 (0.0)	0 (0.0)	7 (0.0)
Negative	1,180 (11.0)	569 (14.7)	28 (5.4)	434 (10.3)	44 (16.5)	2,255 (11.5)
Not tested	9,589 (89.0)	3,296 (85.2)	493 (94.6)	3,789 (89.7)	222 (83.5)	17,389 (88.5)
Mycoplasma						
Positive	6 (0.1)	6 (0.2)	0 (0.0)	2 (0.1)	0 (0.0)	14 (0.1)
Negative	1,214 (11.3)	545 (14.1)	31 (6.0)	506 (12.0)	50 (18.8)	2,346 (11.9)
Not tested	9,553 (88.7)	3,316 (89.8)	490 (94.1)	3,716 (88.0)	216 (81.2)	17,291 (88.0)
Chlamydia*						
Positive	0 (0.0)	0 (0.0)	0 (0.0)	0 (0.0)	0 (0.0)	0 (0.0)
Negative	39 (0.4)	97 (2.5)	4 (0.8)	39 (0.9)	1 (0.4)	180 (0.9)
Not tested	10,734 (99.6)	3,770 (97.5)	517 (99.2)	4,185 (99.1)	265 (99.6)	19,471 (99.1)

*both psittaci and pneumoniae

For the combined group of patients tested for either legionella or mycoplasma (*n* = 2,369) the viral diagnosis was already known at the time of sampling for atypical bacteria for 899 (38.0%). Proportions and associations of patients tested for atypical bacteria are presented in Table 3. Most conspicuous, test frequencies were much higher among patients > 65 years (18.3%) compared to children < 18 years (1.8%) with an aOR of 12.05 (95% CI: 9.73–14.91). In an additional multivariate analysis including vital and laboratory parameters, Regional Hospital Gødstrup was still associated with higher test frequency for atypical bacteria (aOR = 1.99 (95% CI: 1.75–2.26)).

**Table 3:** Proportion and associations with test versus not-tested for atypical pneumonia

		Atypical pneumonia*		Univariate analysis	Sex- and age adjustet analysis
	Variable**	Tested, *n*(%)	Not tested, *n*(%)	OR (95% CI)	aOR (95% CI)
Hospital (city)	Aarhus	494 (10.3)	4,319 (89.7)	1.00	1.00
	Horsens	248 (13.0)	1,662 (87.0)	1.30 (1.11–1.54)	1.06 (0.90–1.25)
	Gødstrup***	861 (19.4)	3,569 (80.6)	2.11 (1.87–2.38)	2.22 (1.96–2.51)
	Randers	316 (8.2)	3,561 (91.8)	0.78 (0.67–0.90)	0.98 (0.84–1.14)
	Silkeborg/Viborg	450 (9.7)	4,171 (90.3)	0.94 (0.82–1.08)	1.21 (1.05–1.39)
Admission year	2019	120 (12.8)	818 (87.2)	1.00	1.00
	2020	178 (9.6)	1,680 (90.4)	0.72 (0.56–0.92)	0.69 (0.53–0.88)
	2021	293 (12.3)	2,134 (87.9)	0.94 (0.75–1.17)	1.14 (0.90–1.44)
	2022	969 (11.4)	7,542 (88.6)	0.88 (0.71–1.07)	0.75 (0.61–0.93)
	2023	656 (12.9)	4,428 (87.1)	1.01 (0.82–1.24)	0.90 (0.73–1.12)
	2024	153 (18.4)	680 (81.6)	1.53 (1.18–1.99)	1.37 (1.05–1.80)
Sex	Female	1,070 (11.4)	8,356 (88.6)	1.00	1.00
	Male	1,299 (12.7)	8,926 (87.3)	1.14 (1.04–1.24)	1.15 (1.05–1.25)
Age	0–17 years	92 (1.8)	4,953 (98.2)	1.00	1.00
	18–65 years	676 (11.6)	5,161 (88.4)	7.05 (5.65–8.80)	7.15 (5.73–8.82)
	65 + years	1,601 (18.6)	7,168 (81.7)	12.02 (9.72–14.88)	12.05 (9.73–14.91)
Vital parameter	Temperature ≤ 38 °C	1,156 (12.1)	8,436 (87.9)	1.00	1.00
	Temperature > 38 °C	1,128 (12.5)	7,860 (87.5)	1.05 (0.96–1.14)	1.23 (1.12–1.34)
	Systolic blood pressure ≥ 90mmHg	2,155 (15.1)	12,130 (84.9)	1.00	1.00
	Systolic blood pressure < 90mmHg	50 (19.2)	211 (80.8)	1.33 (0.98–1.82)	1.46 (1.06–2.00.06.00)
	Respiratory rate ≤ 30/min	1,846 (13.4)	11,901 (86.6)	1.00	1.00
	Respiratory rate > 30/min	447 (8.9)	4.587 (91.1)	0.63 (0.56–0.70)	2.41 (2.12–2.75)
Laboratory Analyses	Leukocyte count ≤ 10.0 × 10^9/ml	1,318 (12.5)	9,225 (89.5)	1.00	1.00
	Leukocyte count > 10.0 × 10^9/ml	906 (21.4)	3,329 (78.6)	1.90 (1.73–2.09)	1.99 (1.81–2.19)
	C-reactive protein ≤ 100 mg/l	1,358 (11.3)	10,667 (88.7)	1.00	1.00
	C-reactive protein > 100 mg/l	865 (31.1)	1,914 (68.9)	3.55 (3.22–3.92)	3.23 (2.92–3.57)
	ALT ≤ 70 U/L****	1,562 (16.6)	7,837 (83.4)	1.00	1.00
	ALT > 70 U/L****	149 (19.3)	623 (80.7)	1.20 (1.00–1.45.00.45)	1.31 (1.09–1.59)
	Creatinine ≤ 105 µmol/l	1,618 (13.9)	10,009 (86.1)	1.00	1.00
	Creatinine > 105 µmol/l	597 (19.7)	2,436 (80.3)	1.52 (1.37–1.68)	1.17 (1.05–1.31)
	Urea ≤ 7 mmol/L	1,234 (13.6)	7,837 (86.4)	1.00	1.00
	Urea > 7 mmol/L	879 (21.1)	3,296 (78.9)	1.69 (1.54–1.86)	1.30 (1.18–1.45)

*Airway samples tested for Legionella and Mycoplasma

**Numbers may not add up to total population due to missing information for some patients

***Before February 2022 hopsitals were located in Herning and Holstebro

***ALT: Alanine transaminase

Empirical clarithromycin was administered to 859 (4.4%) of all the patients, and 531 (61.8%) of these patients already had the result of the viral diagnosis at the time of administration. Median time between admission and first administration of clarithromycin was 5.5 h (interquartile range: 3.3–11.1 h). As presented in Table 4 use of empirical clarithromycin was associated with age > 65 years (aOR 49.65 (95% CI: 24.70–99.80)), and vital signs and laboratory values of more severe disease. Previous allergic reaction to a penicillin was registrered for 12.8% of patients receiving empirical clarithromycin and for 5.3% of patients not did not. After adjusting for sex and age the frequency of CAVE for penicillins were higher in the group receiving empirical clarithromycin (aOR 1.88 (95% CI: 1.52–2.33).

**Table 4:** Usage and associations of empirical clarithromycin

		Empirical Clarithromycin*		Univariate analysis	Sex- and age adjusted analysis
	Variable**	Yes, *n*(%)	No, *n*(%)	OR (95% CI)	aOR (95% CI)
Hospital (city)	Aarhus	157 (3.3)	4,656 (96.7)	1.00	1.00
	Horsens	152 (8.0)	1,758 (92.0)	2.56 (2.04–3.23)	2.05 (1.63–2.58)
	Gødstrup***	175 (4.0)	4,255 (96.0)	1.22 (0.98–1.52)	1.23 (1.63–2.58)
	Randers	177 (4.6)	3,700 (95.4)	1.42 (1.14–1.77)	1.81 (1.45–2.26)
	Silkeborg/Viborg	198 (4.3)	4,423 (95.7)	1.33 (1.07–1.64)	1.71 (1.38–2.12)
Admission year	2019	88 (9.4)	850 (90.6)	1.00	1.00
	2020	87 (4.7)	1,771 (95.3)	0.47 (0.35–0.65)	0.45 (0.33–0.61)
	2021	51 (2.1)	2,376 (97.9)	0.01 (0.15–0.30)	0.24 (0.17–0.35)
	2022	264 (3.1)	8,247 (96.9)	0.31 (0.24–0.40)	0.25 (0.19–0.33)
	2023	292 (5.7)	4,792 (94.3)	0.59 (0.46–0.76)	0.50 (0.39–0.65)
	2024	77 (9.2)	756 (90.8)	0.98 (0.71–1.36)	0.85 (00.61–1.18)
Sex	Female	410 (4.4)	9,016 (95.6)	1.00	1.00
	Male	449 (4.4)	9,776 (95.6)	1.01 (0.88–1.16)	1.00 (0.87–1.15)
Age	0–17 years	8 (0.2)	5,037 (99.8)	1.00	1.00
	18–65 years	210 (3.6)	5,627 (96.4)	23.50 (11.59–47.65)	23.49 (11.58–47.65)
	65 + years	641 (7.3)	8,128 (92.7)	49.65 (24.70–99.81.70.81)	49.65 (24.70–99.80)
Vital parameter	Temperature ≤ 38 °C	383 (4.0)	9,209 (96.0)	1.00	1.00
	Temperature > 38 °C	437 (4.9)	8,551 (95.1)	1.23 (1.07–1.41)	1.48 (1.28–1.71)
	Systolic blood pressure ≥ 90mmHg	783 (5.5)	13,502 (94.5)	1.00	1.00
	Systolic blood pressure < 90mmHg	29 (11.1)	232 (88.9)	2.16 (1.46–3.19)	2.36 (1.58–3.51)
	Respiratory rate ≤ 30/min	627 (4.6)	13,120 (95.4)	1.00	1.00
	Respiratory rate > 30/min	196 (3.9)	4,838 (96.1)	0.85 (0.72–1.00.72.00)	3.31 (2.78–3.95)
Laboratory Analyses	Leukocyte count ≤ 10.0 × 10^9/ml	347 (3.3)	10,196 (96.7)	1.00	1.00
	Leukocyte count > 10.0 × 10^9/ml	465 (11.0)	3,770 (89.0)	3.62 (3.14–4.19)	3.75 (3.24–4.34)
	C-reactive protein ≤ 100 mg/l	348 (2.9)	11,677 (97.1)	1.00	1.00
	C-reactive protein > 100 mg/l	465 (16.7)	2,314 (83.3)	6.74 (5.83–7.80)	6.03 (5.21–6.99)
	ALT ≤ 70 U/L****	556 (5.9)	8,843 (92.7)	1.00	1.00
	ALT > 70 U/L****	75 (9.7)	697 (90.3)	1.71 (1.33–2.20)	1.96 (1.52–2.54)
	Creatinine ≤ 105 µmol/l	561 (4.8)	11,066 (95.2)	1.00	1.00
	Creatinine > 105 µmol/l	252 (8.3)	2,781 (91.7)	1.79 (1.53–2.09)	1.38 (1.17–1.62)
	Urea ≤ 7 mmol/L	365 (4.0)	8,706 (96.0)	1.00	1.00
	Urea > 7 mmol/L	421 (10.1)	3,754 (89.9)	2.67 (2.31–3.09)	2.06 (1.77–2.41)

*Empirical Clarithromycin: prescribed with 48 h of admission and before the test result of atypical pneumonia (legionella and mycoplasma)

**Numbers may not add up to total population due to missing information for some patients

***Before February 2022 hopsitals were located in Herning and Holstebro

***ALT: Alanine transaminase

Patients with an atypical bacterial co-infection had lower median age (53.0 years) than those testing negative (73.1 years, *p* < 0.01). There was no difference in median leukocyte count (10.1 versus 9.0, *p* = 0.81) or median CRP levels (43 vs. 71, *p* = 0.81).

Of the 21 patients that tested positive for an atypical bacteria (either legionella or mycoplasma) 17 (81.0%) had not received empirical clarithromycin. The 17 patients did not receive any other empirical antibiotic treatment covering atypical bacteria.

## Discussion

In this large regionwide cohort study we included 19,651 patients admitted with a viral respiratory infection. Only 21 patients tested positive for atypical bacterial co-infection, corresponding to 0.1% of those tested. Empirical clarithromycin was administered to 859 (4.4%) patients - still 81.0% of patients with atypical bacterial co-infection were missed before the result of diagnostic test was available. Both testing frequency and empirical clarithromycin was associated with year, age, and more severe disease presentation.

Only few other studies have focused specifically on atypical bacterial co-infections among patients with a viral respiratory tract infection. In a Korean study from the period 2010–2016 [14] Kim et al. enrolled patients with influenza and radiographic pneumonia, but excluded patients with non-mycoplasma bacterial co-infection. From a total cohort of 4,465 influenza patients 244 fulfilled the eligibility criteria, and the investigators found 41 (16.8%) patients co-infected with *Mycoplasma pneumonia*. A more recent study from India included 194 patients with SARS-CoV-2 and reported 10 (5.2%) patients co-infected with *Mycoplasma pneumonia* [15]. However in both studies a large proportion of mycoplasma diagnoses were based on mycoplasma-IgM titres and not confirmed by PCR. As mycoplasma IgM levels may persist for months after initial infection both studies may have overestimated actual mycoplasma cases [16]. Other explanations for the differences in the proportion of mycoplasma cases could be selection bias due to enrollment of patients at tertiary centers, age of the study population as mycoplasma may be more frequent in younger individuals, and timing of the studies related to concurring mycoplasma epidemics.

Other studies reporting the heterogenicity of bacterial co-infections have found only few patients co-infected with an atypical bacteria [6, 17–19]. Compared to these studies the use of urine antigen tests detecting *Streptococcus pneumonia* and *Legionella pneumophila* was low (121 patients tested = 0.6%). In our region the urine antigen test is not generally considered cost-effective and is therefore reserved for patients at intensive care wards. Restricting the urine antigen test to patients in the intensive care ward may have led to a few missed cases. Still, we conclude that atypical bacterial co-infections in patients admitted with these respiratory tract viruses are rare.

A major strength of this study is the regionwide cohort design including patients admitted to both regional and the university hospital, in the Central Denmark Region. Thus, there is very little risk of patient selection bias compared to single-hospital studies or studies from reference hospitals involved in an academic network. The systematic approach included patients of all ages and we found relevant differences in the diagnostic and antimicrobial approaches between different hospitals and for pediatric and adult patient groups.

Because this was a retrospective cohort study, there was no study related intervention for a systematic approach to diagnostic tests and diagnostic test prescriptions relied on the physicians caring for the individual patients. We found about ~ 10% of the patients had been tested for at least one of the atypical bacterial pathogens within the 7-day period after admittance. In theory, some patients with an atypical bacterial co-infection could have been missed and our finding would thereby be an underreporting of the true number of atypical bacterial co-infections. However, we consider the risk minor and extending the diagnostic window would open up for registration of hospital-acquired infections which was not the aim of this study [20]. Also, we reported the actual number of diagnostic tests performed and not the number of test prescriptions by physicians. Patients with a viral respiratory tract infection may not be able to produce a relevant sample from the deep airways which also explains the slightly higher test rate for mycoplasma which in contrast to Legionella may be performed on a throat swap.

Use of diagnostic tests for atypical bacterial co-infections and prescribing empirical clarithromycin are not mentioned in guidelines for viral respiratory tract infections in Denmark [21] or internationally [22, 23]. Yet, our study shows that in daily clinical work these practices are often done even though the result of viral testing was most often available when sampling patient for atypical bacteria and administering clarithromycin. This practice is likely inspired by guidelines for handling community-acquired pneumonia [8, 9] where both are recommended in case of severe pneumonia. During the study period the regional guideline for treating community acquired pneumonia specifically mentioned the macrolide Clarithromycin for atypical coverage in patients with severe pneumonia.

Atypical pathogens are proportionally much more common in the patient group with community-acquired pneumonia [24]. Moreover mycoplasma occurs in epidemics with years between, and since 2023 there has been a significant increase in mycoplasma cases [25]. This may also explain the higher test rate in the end of our study period due to increased awareness among clinicians. The unusual epidemic patterns of both mycoplasma and the respiratory tract viruses following the emergence of COVID may have decreased generalizability of our study findings. Most likely many of the early COVID cases were more severe due to lack of available treatment options and vaccines. Correspondingly, we demonstrated varying test frequency for atypical bacteria and use of clarithromycin during the study period.

Our study shows that legionella, mycoplasma and chlamydial co-infection is very rare in patients admitted with viral respiratory tract infection. Ideally, characterizing patients with an atypical bacterial co-infection could identify features distinguishing them from other patients. This would allow for a more precise algorithm for ordering diagnostic tests and prescribing relevant empirical treatment. We found that patients with an atypical bacterial co-infection had a slightly lower median age, but there was no difference in the inflammation marker CRP or leukocyte count. Further characterization of the patients with a positive test for legionella or mycoplasma was not performed due to the low event rate and thereby risk of recognisability of individuals. Based on our results we advise against routine use of both testing for atypical bacterial co-infection and prescription of empirical treatment of atypical bacterial co-infection. Exceptions would be severely sick patient, patients with an abnormal course of illness, a history of potential exposure to atypical bacteria, or during an epidemic wave of mycoplasma.

Clarithromycin has been suggested as an immune-modulating agent that potentially could be beneficial when treating respiratory viral infections [26–28]. In our study we considered the use of clarithromycin in the global initiative to combat antimicrobial resistance and from this perspective its usage should be limited. Fortunately macrolide-resistant *Mycoplasma pneumonia* is rare in Denmark [29], but other regions of the world have to use more broad spectrum antibiotics such as doxycycline due to higher levels of resistance. The potential role of clarithromycin as an immune-modulator remains to be clarified.

## Data Availability

The datasets analysed during the current study are not publicly available due to patient confidentiality.

## Declarations

### Access to data

BLH and JR had full access to data and are guarantors for the data.
